# Atypical Protein Phosphatases: Emerging Players in Cellular Signaling

**DOI:** 10.3390/ijms14034596

**Published:** 2013-02-26

**Authors:** Daichi Sadatomi, Susumu Tanimura, Kei-ichi Ozaki, Kohsuke Takeda

**Affiliations:** 1 Division of Cell Regulation, Graduate School of Biomedical Sciences, Nagasaki University, 1-14 Bunkyo-machi, Nagasaki 852-8521, Japan; E-Mails: sad.atom.66038@gmail.com (D.S.); tani1211@nagasaki-u.ac.jp (S.T.); kozak@nagasaki-u.ac.jp (K.O.); 2 Precursory Research for Embryonic Science and Technology (PRESTO), Japan Science and Technology Agency (JST), 4-1-8 Honcho Kawaguchi, Saitama 332-0012, Japan

**Keywords:** protein phosphatase, EYA, Sts, PGAM5, mitochondria

## Abstract

It has generally been considered that protein phosphatases have more diverse catalytic domain structures and mechanisms than protein kinases; however, gene annotation efforts following the human genome project appeared to have completed the whole array of protein phosphatases. Ser/Thr phosphatases are divided into three subfamilies that have different structures from each other, whereas Tyr phosphatases and dual-specificity phosphatases targeting Tyr, Ser and Thr belong to a single large family based on their common structural features. Several years of research have revealed, however, the existence of unexpected proteins, designated here as “atypical protein phosphatases”, that have structural and enzymatic features different from those of the known protein phosphatases and are involved in important biological processes. In this review, we focus on the identification and functional characterization of atypical protein phosphatases, represented by eyes absent (EYA), suppressor of T-cell receptor signaling (Sts) and phosphoglycerate mutase family member 5 (PGAM5) and discuss their biological significance in cellular signaling.

## 1. Introduction

**Figure 1:**
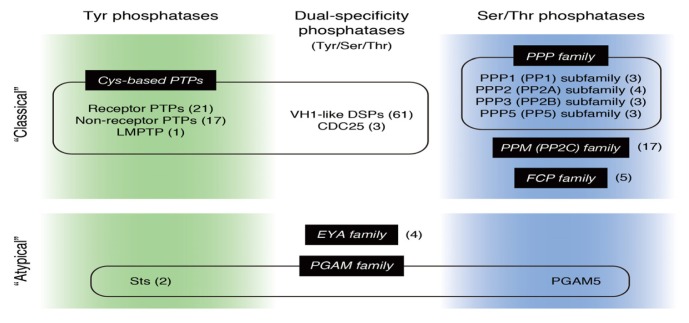
“Classical” and “atypical” protein phosphatases in humans. Protein Tyr phosphatases and dual-specificity phosphatases constitute a single large family of Cys-based phosphatases, whereas protein Ser/Thr phosphatases are divided into three subfamilies, the phosphoprotein phosphatase (PPP), protein phosphatase Mg^2+^- or Mn^2+^-dependent (PPM) (PP2C) and FCP families. Eyes absent (EYA), suppressor of T-cell receptor signaling (Sts) and phosphoglycerate mutase family member 5 (PGAM5) are recently characterized “atypical” protein phosphatases that do not belong to the “classical” protein phosphatase families. Numbers in parentheses indicate the number of genes encoding each family member. Many members of the PPP family consist of multiple subunits; therefore, only the genes encoding the catalytic subunits are counted. PTP, protein Tyr phosphatase; LMPTP, low molecular weight PTP; VH1-like DSP, Vaccinia virus gene H1-like dual-specificity phosphatase; PPP, phosphoprotein phosphatase; PPM, metal-dependent protein phosphatase; FCP, TFIIF-associating component of RNA polymerase II carboxy-terminal domain phosphatase.

Protein kinases have highly conserved catalytic domain structures, irrespective of their substrate specificity, and all protein kinases were annotated relatively soon after the human genome project was completed [1]. Similar approaches have been used to annotate all of the protein phosphatases in the human genome, although they have more diverse catalytic domain structures than those of protein kinases [2,3]. Tyr phosphatases and dual-specificity phosphatases targeting Tyr, Ser and Thr constitute a single large family based on their common structural features. These proteins use an essential Cys residue as a nucleophile that forms a thiol-phosphate intermediate during catalysis [4]. Ser/Thr phosphatases are divided into three subfamilies that have different structures, the phosphoprotein phosphatase (PPP) and metal-dependent protein phosphatase (PPM) metalloenzyme families and the TFIIF-associating component of RNA polymerase II carboxy-terminal domain phosphatase (FCP) Asp-based phosphohydrolase family [5] (Figure 1). In spite of a consensus that these phosphatases cover all the protein dephosphorylation events, accumulating evidence has shown that several other proteins function as protein phosphatases and have structural and enzymatic features different from those of the known protein phosphatases. Because the phosphatase activities of these new phosphatases have been shown to have *in vitro* potential and to be involved in important biological processes, we here designate them as atypical protein phosphatases and review their unique molecular and functional features, with particular focus on their roles in cellular signaling.

## 2. EYA: A New Type of Dual-Specificity Protein Phosphatase

Eyes absent (EYA) was originally identified as a novel nuclear protein in *Drosophila*[6]. Because EYA lacks a recognizable DNA-binding motif, but operates genetically and biochemically in close synergy with the Six family transcription factor, sine oculis (SO), and the nuclear cofactor, Dachshund (Dac), EYA is proposed to function as a transcriptional cofactor. In fact, EYA, SO and Dac constitute a synergistic nuclear complex that is necessary and sufficient for eye development in *Drosophila*[7]. Orthologs of EYA have been identified in many species, including worms, fish and mammals. In mammals, there are four isoforms, EYA1–EYA4. Mutation in human *EYA1* causes autosomal dominant genetic disorders characterized by craniofacial abnormalities, hearing loss and kidney defects [8,9]. Consistent with these effects, EYA1-deficient mice exhibit renal abnormalities and a conductive hearing loss [10]. EYA3-deficient mice have minor defects in respiratory, muscle and heart functions and locomotion [11]. EYA4-deficient mice exhibit severe hearing deficits and develop otitis media with effusion [12], which may resemble the effects of the *EYA4* mutations, which cause sensorineural hearing loss accompanied by cardiomyopathy in humans [13–16].

### 2.1. Protein Tyr Phosphatase Activity of EYA

**Figure 2:**
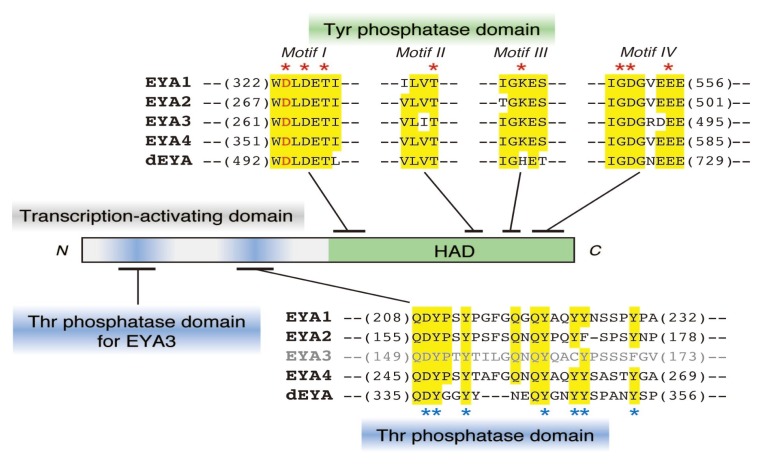
The primary structure of EYA. The structural features of the EYAs and the amino acid sequences of the phosphatase domains of mouse EYA1–EYA4 and *Drosophila* EYA (dEYA) are shown. Red asterisks indicate the amino acid residues critical for Tyr phosphatase activity. The first Asp in motif I, highlighted by red, acts as a catalytic center, and motif IV contributes to the retention of divalent ions, such as Mg^2+^[7]. The Thr phosphatase domain of EYA3 resides near the *N*-terminal region, whereas that of EYA4, dEYA and (most likely) EYA1 and EYA2 is located near the C-terminal region, within the transcription-activating domain. Among the seven residues, indicated by blue asterisks, the combinational mutation of the former three (DYY) or latter four (YYYY) residues abolishes the Thr phosphatase activity, but not the Tyr phosphatase activity, of EYA4 [24]. Residues that are identical among four or all five proteins are highlighted by yellow. Numbers in the parentheses indicate the amino acid position in each sequence.

In 2003, three independent research groups simultaneously reported that EYA is not only a transcription factor, but also a protein Tyr phosphatase [17–19]. Whereas the catalytic activity of conventional Tyr phosphatases depends on a Cys residue in the catalytic core, as mentioned above, the catalytic activity of EYA depends on an Asp residue in the C-terminal haloacid dehalogenase (HAD) domain and requires divalent ions, such as Mg^2+^, which are unique features among protein Tyr phosphatases (Figure 2). This enzymatic activity is also required for the ability of EYA to promote eye development in *Drosophila*[17,18], as well as to regulate mammalian organogenesis [19].

**Figure 3:**
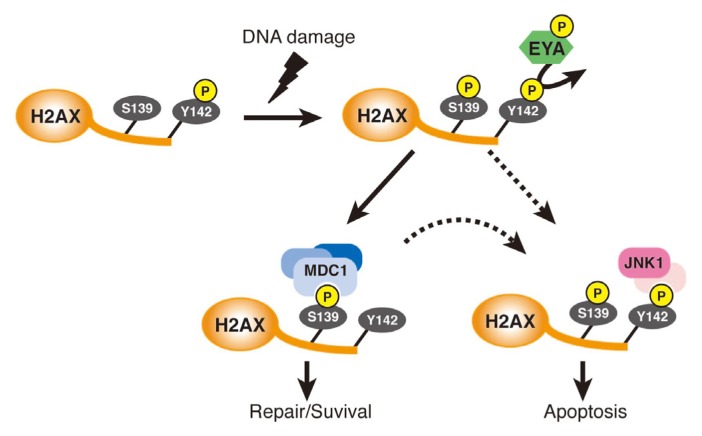
Regulation of the phosphorylation of H2AX and cellular responses to DNA damage. Upon DNA damage, EYA dephosphorylates Tyr142 of H2AX, facilitating phosphorylation of Ser139 of H2AX. If the damage can be repaired, a series of DNA repair factors, including MDC1, are recruited to H2AX. If the damage cannot be repaired, however, an apoptosis-inducing complex, including JNK1, is formed on H2AX, most likely through persistent phosphorylation or re-phosphorylation of Tyr142, indicated by dashed arrows.

Nuclear phosphorylation of the histone variant, H2AX, is a crucial component in DNA damage repair. It has recently been shown that DNA damage induces the interaction of EYA with H2AX through phosphorylation of EYA by the DNA-damage-response protein kinases, ataxia telangiectasia mutated (ATM) and ataxia-telangiectasia and Rad-3-related (ATR) [20–22]. EYA subsequently dephosphorylates Tyr142 in the *C*-terminal tail of H2AX, which is basally phosphorylated under unstimulated conditions [22,23] (Figure 3). The dephosphorylation of Tyr142 triggers phosphorylation of the nearby Ser139, facilitating the recruitment of DNA repair factors, including mediator of DNA damage check point protein 1 (MDC1), meiotic recombination 11 (MRE11) and Rad50, to H2AX. When Tyr142 is not dephosphorylated even under DNA damage-inducing conditions, the repair factors do not bind to H2AX, eventually leading to apoptosis through the recruitment of proapoptotic proteins, including c-Jun *N*-terminal kinase 1 (JNK1) to H2AX [22]. These findings strongly suggest that the Tyr phosphatase activity of EYA is an active determinant of repair and survival *versus* apoptosis in response to DNA damage.

Consistent with the finding that the overexpression of some of the EYA isoforms correlates with tumor growth and increased metastasis in various cancers [25–27], it has been reported that the Tyr phosphatase activity of EYA promotes tumor cell migration, invasion and transformation, concomitant with alterations in the actin cytoskeleton and activation of Rac and Cdc24, which are members of the Rho-GTPase family that play important and diverse roles in the reorganization of the actin cytoskeleton [28]. It has also been reported that the Tyr phosphatase activity of EYA is pro-angiogenic, as demonstrated by the attenuation of cell motility and capillary morphogenesis of human umbilical vein endothelial cells (HUVECs) upon EYA knockdown [29]. The requirement of Tyr phosphatase activity for the pro-angiogenic activity of EYA was clearly demonstrated by various assays, including the *in vivo* vasculature assay in zebrafish, using the uricosuric agents, benzbromarone and benzarone, which were recently discovered to be specific inhibitory compounds against the Tyr phosphatase activity of EYA. Thus, the Tyr phosphatase activity of EYA could be a therapeutic target for inhibiting tumor invasion, angiogenesis and metastasis, as well as various vasculopathies.

### 2.2. EYA Also Functions as a Thr Phosphatase

More recently, it has unexpectedly been found that EYA functions also as a protein Thr phosphatase [24]. Intriguingly, this Thr phosphatase activity appears to be exerted by the *N*-terminal transcription-activating domain, whereas the Tyr phosphatase activity relies on the *C*-terminal HAD domain (Figure 2). In fact, optimal conditions for catalysis are different for the two phosphatase activities. The Tyr phosphatase has optimal activity under acidic conditions and requires divalent ions, whereas the Thr phosphatase functions under basic conditions without a requirement for metal ions. This property, that the dual substrate specificity is exerted by two independent domains within a molecule, is unique, because the catalytic activity of the previously known dual-specificity phosphatases completely relies on a single catalytic domain. Nevertheless, the catalytic mechanisms of EYA Thr phosphatases appear to vary among the EYA isoforms, because the catalytic domain of EYA3 has recently been found to be different from that of other EYAs, though both are within the *N*-terminal transcription-activating domain [30]. Further analyses will thus be required for a full understanding of the catalytic mechanism of the Thr phosphatase activity of EYA.

Importantly, the Thr phosphatase activity, but not the Tyr phosphatase activity, of EYA4 plays a critical role in innate immunity [24]. EYA4 enhances the innate immune response to viruses, including Newcastle disease virus and vesicular stomatitis virus, and non-microbial danger signals, such as the undigested DNA of apoptotic cells, through upregulation of the expression of interferon-β (IFN-β) and C-X-C motif chemokine 10 (CXCL10). EYA4 appears to act as a Thr phosphatase in a large protein complex consisting of various signaling molecules required for the innate immune response, such as MAVS/IPS-1-1/VISA/Cardif, STING/TMEM173 and NLRX1, although the dephosphorylation substrates of EYA4 have not been identified.

A role for EYA in innate immunity has been shown to be conserved among species [31]. *Drosophila* EYA also has the *N*-terminal Thr and C-terminal Tyr phosphatase domains. Similar to mammalian EYA4, the Thr phosphatase activity, but not the Tyr phosphatase activity, of EYA is responsible for the innate immune response to DNA. EYA associates with the nuclear factor-κB (NF-κB)-like factor, Relish, and its kinase, IκB kinase (IKKβ), both of which are components of the Immune Deficiency (IMD) pathway [32] and induce the IMD pathway-dependent expression of the antimicrobial peptide gene *Attacin-A*. This inducing activity of EYA toward the *Attacin A* gene does not depend on its transcriptional binding partner SO, suggesting that the Thr phosphatase activity, rather than the transcriptional activity, of EYA plays a pivotal role in the innate immune response to DNA in *Drosophila*.

## 3. Sts-1 and Sts-2: Members of the PGAM Family that Function as Protein Tyr Phosphatases

Suppressor of T-cell receptor signaling-1 (Sts-1), also known as p70 or T-cell ubiquitin ligand 2 (TULA2), was originally identified as a protein that binds Janus kinase 2 (Jak2), a protein Tyr kinase involved in a specific subset of cytokine receptor signaling pathways [33]. Sts-1 was also identified later as a binding partner of the ubiquitin ligase, c-Cbl, which plays a critical role in attenuating receptor Tyr kinase (RTK) signaling by inducing ubiquitination of RTKs and promoting their sorting for endosomal degradation [34]. Sts-2, also known as TULA or UBASH3A, a protein highly homologous to Sts-1, was also identified as a binding partner of c-Cbl [34,35].

**Figure 4:**
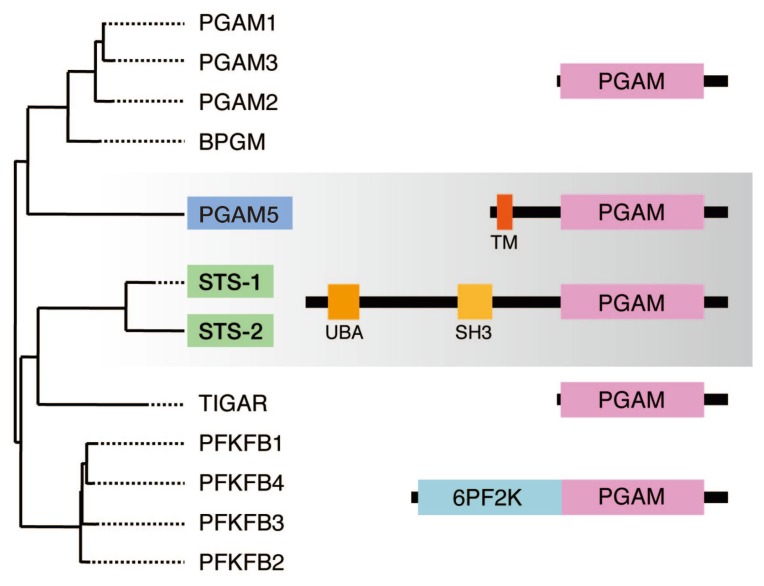
The phosphoglycerate mutase (PGAM) family. Human PGAM family members and their domain structures are shown. A phylogenetic tree was created by the alignment of PGAM domains using the ClustalW program. BPGM, 2,3-bisphosphoglycerate mutase; TIGAR, TP53-induced glycolysis and apoptosis regulator; PFKFB, 6-phosphofructo-2-kinase/fructose-2,6-bisphosphatase.

Sts-1 and Sts-2 are members of the phosphoglycerate mutase (PGAM) family (Figure 4). PGAM is the founding member of this family and an evolutionarily conserved enzyme of intermediary metabolism that converts 3-phosphoglycerate to 2-phosphoglycerate in glycolysis [36]. Members of this family share a common catalytic domain, designated as the PGAM domain, and function as phosphotransferases and/or phosphohydrolases with small molecular substrates. The catalytic core of the PGAM domain centers on a His residue that acts as a phospho-acceptor during catalysis. These biochemical and structural features are shared with the His acid phosphatase family of enzymes, and it has recently been proposed that these two families can be integrated into a His phosphatase superfamily [37]. Sts-1 and Sts-2 have this domain in their C-terminal regions. In addition, they have the ubiquitin-associated (UBA) and Src-homology 3 (SH3) domains, through which they bind ubiquitin and c-Cbl, respectively [34,35,38]. Whereas Sts-1 is ubiquitously expressed in mammalian tissues, Sts-2 is preferentially expressed in cells and tissues of the hematopoietic systems [33,35,39,40].

### 3.1. His-Based Tyr Phosphatase Activity of Sts-1/2 and TCR Signaling

Signaling through the multisubunit T-cell antigen receptor (TCR) precisely controls the development and function of T-lymphocytes, which play a critical role in the recognition and elimination of foreign pathogens [41]. Whereas mice deficient either in Sts-1 or Sts-2 exhibited no obvious abnormalities, T-cells from mice lacking both Sts-1 and Sts-2 were hyperresponsive to TCR stimulation, as indicated by increased phosphorylation and activation of the Tyr kinase Zap-70, which is recruited to the TCR complex and plays a critical role in propagating signals initiated by TCR engagement [33,39,42]. In addition, hyperactivation of signaling molecules downstream of the TCR and an increase in cytokine production were observed in Sts-1/2-deficient T-cells. Consistent with this result, Sts-1/2-deficient mice exhibited increased susceptibility to experimental autoimmune encephalomyelitis (EAE), a mouse model for multiple sclerosis [39].

Later analysis revealed that Sts-1 has an intrinsic protein Tyr phosphatase activity associated with the C-terminal PGAM domain [43]. Through its activity, Sts-1 dephosphorylates Zap-70 and other molecules downstream of the TCR and, thus negatively regulates the TCR signaling pathways. The two His and two Arg residues that are conserved among the PGAM family members are also conserved in the PGAM domain of Sts-1, and mutation of either or both of the two His residues abolishes the phosphatase activity of Sts-1, indicating that Sts-1 is a novel His-based protein Tyr phosphatase, the catalytic structure of which is different from that of the previously known Cys-based protein Tyr phosphatases. Importantly, hyperresponsiveness of Sts-1/2-deficient T-cells was reduced by reconstitution of the wild-type Sts-1, but not by a mutant Sts-1 in which the catalytic His was substituted by Ala. These findings demonstrate that the phosphatase activity of Sts-1 plays a critical role in the control of TCR signaling.

In contrast to Sts-1, the intrinsic phosphatase activity of Sts-2 is known to be quite low, although its PGAM domain is highly homologous to that of Sts-1 and retains the conserved catalytic core of two His and two Arg [43,44]. Sts-2 thus had been thought to have a very narrow substrate spectrum or not to function as a phosphatase within the cell. A recent investigation has revealed, however, that Sts-2 has definite, albeit weak, phosphatase activity; Sts-2 hydrolyzes phosphorylated fluorescein analogues, including O-methylfluorescein phosphate (OMFP) and fluorescein diphosphate (FDP), with significantly greater efficiency than it hydrolyzes the conventionally used phosphatase substrate, para-nitrophenyl-phosphate (pNPP) [45]. A careful comparison among T-cells deficient in Sts-1 or Sts-2 or both has also revealed that Sts-2 indeed regulates the level of Tyr phosphorylation on target molecules, including Zap-70 within T-cells. Taken together, the Tyr phosphatase activities of Sts-1 and Sts-2 appear to regulate TCR signaling cooperatively.

### 3.2. Regulation of Protein Tyr Kinase Signaling by Sts-1/2

As mentioned above, Sts-1 and Sts-2 were identified as interacting proteins of c-Cbl. They bind c-Cbl through their SH3 domains and are recruited to epidermal growth factor (EGF) receptor (EGFR) with c-Cbl upon EGF stimulation. They then suppress the internalization and subsequent degradation of EGFR, enhancing the downstream signaling of EGFR [34,35]. Although Sts-1 and Sts-2 both facilitate stabilization of EGFR, their mechanisms of action appear to differ. Sts-1 dephosphorylates EGFR at multiple phosphorylated Tyr residues, including those involved in the recognition of EGFR by c-Cbl, and, thereby, stabilizes EGFR, whereas the UBA domain of Sts-1 does not contribute significantly to receptor stabilization [46]. In contrast, Sts-2 appears to stabilize EGFR by masking the c-Cbl-mediated ubiquitination site on EGFR by the UBA domain of Sts-2 and suppressing ubiquitination-dependent endocytosis. Furthermore, Sts-2 has been proposed to facilitate degradation of c-Cbl through its autoubiquitination [35,38].

In addition to stabilizing EGFR, Sts-2 inhibits the uptake of transferrin and low-density lipoprotein (LDL), suggesting that Sts-2 negatively regulates clathrin-dependent endocytosis in general [47]. Furthermore, it has been shown that Sts-2 binds dynamin, a well-characterized GTPase responsible for endocytosis, and inhibits clathrin-independent, but dynamin-dependent, endocytosis. Thus, Sts-2 appears to regulate a wide range of endocytotic activity. In contrast, the generality of the endocytotic regulation by Sts-1 has remained elusive, although Sts-1 has the potential to regulate another RTK, the platelet-derived growth factor (PDGF) receptor [34].

Syk, a protein Tyr kinase that plays a crucial role in lymphoid signaling, is another dephosphorylation target of Sts-1 [48,49]. Recently, a role for the physical and functional interaction between Sts-1 and Syk in mast cell degranulation has been proposed [50]. Mast cell stimulation during allergic inflammation is mediated by the high affinity immunoglobulin E (IgE)-binding receptor (FcɛRI). Activation of FcɛRI resulted in the Tyr phosphorylation of Syk, and upon this phosphorylation, Sts-2 interacted with Syk. The knockdown of Sts-2 with siRNA increased the FcɛRI-induced Tyr phosphorylation of Syk concomitant with the enhancement of the receptor-induced degranulation and activation of the nuclear factor for T-cell activation (NFAT) and NF-κB in mast cells, suggesting that Sts-1 functions as a negative regulator of FcɛRI signaling in mast cells. In summary, Sts-1 and Sts-2 play critical roles at multiple steps of signaling through protein Tyr kinases

## 4. PGAM5: Another Member of the PGAM Family that Functions as a Protein Ser/Thr Phosphatase

PGAM5 is another member of the PGAM family and possesses the PGAM domain and an N-terminal transmembrane (TM) domain (Figure 4). PGAM5 was first identified and reported as an interacting protein of the anti-apoptotic factor Bcl-x_L_[51]. PGAM5 was later reported as an interacting protein of Keap1, a BTB-Kelch substrate adaptor protein for a Cul3-dependent ubiquitin ligase complex that is a sensor for thiol-reactive chemopreventive compounds and oxidative stress [52]. PGAM5 contains a conserved NXESGE motif (amino acids 77–82 of human PGAM5) that binds to the substrate-binding pocket in the Kelch domain of Keap1 [53,54], and Keap1-dependent ubiquitination of PGAM5 leads to proteasome-dependent degradation of PGAM5. Furthermore, it has been reported that PGAM5 is localized to the mitochondria through its TM domain, where it forms a ternary complex with Keap1 and the Nrf2 transcription factor [55].

### 4.1. PGAM5 is a His-Based Protein Ser/Thr Phosphatase that Activates the Stress-Activated MAP Kinase Pathways

Independently of the above-mentioned studies, our group identified PGAM5 as an interacting protein of a stress-activated protein kinase apoptosis signal-regulating kinase 1 (ASK1) in a pull-down-based proteomic analysis [56]. ASK1 is an upstream regulator of the stress-activated JNK and p38 MAP kinase pathways and plays critical roles in cellular responses to oxidative stress, endoplasmic reticulum (ER) stress, inflammatory cytokines and microbial infections [57,58]. Overexpression of PGAM5 activated the ASK1-JNK and ASK1-p38 pathways, and knockdown of PGAM5 reduced the basal ASK1 activity in some cultured cells, suggesting that PGAM5 is an activator of ASK1. Because, similar to ASK1, PGAM5 is ubiquitously expressed in various tissues [59], PGAM5 may be a general regulator of ASK1 activity. Nevertheless, considering that PGAM5 is localized to the mitochondria, while ASK1 is thought to exist diffusely within the cytosol, PGAM5 may regulate only a fraction of the ASK1 that exists at or near the mitochondria.

During the investigation of the mechanism of PGAM5-induced ASK1 activation, PGAM5 was found to dephosphorylate the basally phosphorylated C-terminal region of ASK1 directly [56]. This phosphatase activity of PGAM5 was abolished when the putative catalytic center, His105, was mutated. Consistent with this result, bacterially generated recombinant PGAM5 dephosphorylated phospho-Ser and phospho-Thr peptides, but not phospho-Tyr peptides, depending on its His105. These findings demonstrate that PGAM5 is a novel His-based protein Ser/Thr phosphatase. Currently, it is proposed that PGAM5 activates ASK1 by dephosphorylating the inactivating phosphorylation site(s) of ASK1.

### 4.2. PGAM5, Together with Sts-1/2, Constitutes a Novel Family of Protein Phosphatases

Similar to Sts-1/2, the catalytic structure and probable catalytic mechanism of PGAM5 are different from those of the classical Ser/Thr phosphatases described above. Intriguingly, the primary structures of the catalytic domains of Sts-1/2 and PGAM5 are highly homologous to each other, indicating that PGAM5 and Sts-1/2 constitute a novel and unique family of protein phosphatases with distinctly different substrate specificities, in spite of their similar structures. Therefore, Tyr-specific and Ser/Thr-specific protein phosphatases do not necessarily possess different structures from each other.

The biochemical and structural features of the PGAM family members are also shared with those of the His acid phosphatase family of enzymes as described above [37]. A fraction of one of the His acid phosphatase family members, prostatic acid phosphatase (PAcP), is expressed intracellularly and has been reported to dephosphorylate Tyr residues on the ErbB-2 proto-oncogene product and regulate prostate cancer cell growth [60]. Thus, there is a possibility that the His phosphatase superfamily still includes more members that function as unique protein phosphatases.

### 4.3. PGAM5 May Be Involved in the Mitochondrial Quality Control System

**Figure 5:**
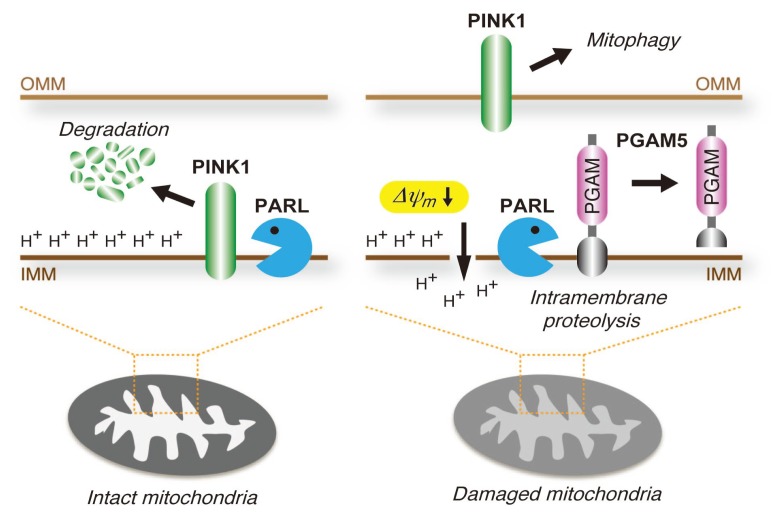
Mitochondrial membrane potential-dependent cleavage of PGAM5 by presenilin-associated rhomboid-like (PARL). PGAM5 is predominantly localized to the inner mitochondrial membrane (IMM) and is cleaved in its *N*-terminal transmembrane (TM) domain in response to a loss of mitochondrial membrane potential (Δψ*_m_*). PTEN-induced kinase 1 (PINK1) is another substrate of PARL, but is cleaved and degraded only in intact mitochondria. Upon the loss of *Δψ_m_*, PARL appears to switch its substrate from PINK1 to PGAM5, resulting in stabilization of PINK in the outer mitochondrial membrane (OMM) and subsequent mitophagy.

It has recently been demonstrated that PGAM5 is predominantly localized to the inner mitochondrial membrane (IMM) with the *C*-terminal PGAM domain facing the intermembrane space [61]. Interestingly, PGAM5 is cleaved in its TM domain in response to a loss of mitochondrial membrane potential (Δψ*_m_*), a hallmark of mitochondrial dysfunction or dysregulation, suggesting that PGAM5 monitors mitochondrial conditions (Figure 5). Furthermore, this unique intramembrane proteolysis is mediated by the IMM-resident Rhomboid serine protease, presenilin-associated rhomboid-like (PARL). PARL has recently been focused on as an important regulator of the PTEN-induced kinase 1 (PINK1), a mitochondrial Ser/Thr protein kinase known as a gene product responsible for early-onset autosomal recessive Parkinson’s disease; PARL mediates rapid and constitutive degradation of PINK1 in the IMM under steady-state conditions [62–64]. Upon Δψ*_m_* loss, however, PINK1 escapes from the cleavage by PARL and stabilizes in the outer mitochondrial membrane (OMM), triggering mitophagy, which selectively eliminates damaged mitochondria by autophagy and, thus, is an important component of the mitochondrial quality control system [65–67]. Taken together with the finding that PARL dissociated from PINK1 and reciprocally associated with PGAM5 upon Δψ*_m_* loss [61], PARL appears to mediate differential cleavage of PGAM5 and PINK1, depending on Δψ*_m_*. Considering that PGAM5 itself responds to Δψ*_m_* loss, PGAM5 may also be involved in the mitochondrial quality control system.

This possibility is supported by the recently found genetic interaction between PGAM5 and PINK1 in *Drosophila*. PGAM5 is highly conserved from *Drosophila* to mammals and *Drosophila* PGAM5 (dPGAM5) retains protein Ser/Thr phosphatase activity [56]. Muscle degeneration, motor defects and the shorter lifespan of *Drosophila* PINK1 (dPINK1)-deficient flies, all of which appear to mimic Parkinson’s disease and to be attributed to mitochondrial degeneration, were suppressed in the double mutants deficient in both dPINK1 and dPGAM5 [68]. These genetic data suggest that dPGAM5 exerts deleterious effects on flies in the context of loss of dPINK1. More analysis of the functional interaction between PGAM5 and PINK1 would reveal the role for PGAM5 in the mitochondrial quality control and possibly in the pathogenesis of Parkinson’s disease.

### 4.4. PGAM5 Regulates Cell Death in a Context-Dependent Manner

Recent findings suggest that PGAM5 has both anti- and pro-apoptotic functions. Flies deficient in dPGAM5 exhibited increased vulnerability to heat shock stress [69]. Selective knockdown of dPGAM5 in the mushroom body, a brain structure that plays a central role in higher brain functions, such as olfactory learning and memory, is sufficient for vulnerability to heat shock stress, indicating that dPGAM5 in the mushroom body plays a critical role in the whole-body response to heat shock stress. Importantly, phosphatase-inactive dPGAM5 could not rescue this vulnerability, indicating that the role for dPGAM5 in heat shock response depends on its phosphatase activity. Moreover, after heat shock treatment, apoptotic cells are detected in the mushroom body of dPGAM5-deficient flies, but not in wild-type flies, indicating that dPGAM5 protects flies against heat shock stress by preventing apoptosis in the mushroom body. Although the detailed molecular mechanisms by which dPGAM5 prevents apoptosis after heat shock stress are not known, there is a possibility that dPGAM5 senses heat shock stress-induced mitochondrial defects, such as the accumulation of heat-damaged proteins within mitochondria, and transduces signals from mitochondria to other cellular compartments.

In contrast, it has been reported that PGAM5 bridges Keap1 and Bcl-x_L_ and facilitates the Keap1-E3 ligase complex-mediated degradation of Bcl-x_L_ and subsequent apoptosis, suggesting the pro-apoptotic role of PGAM5 [70]. More recently, the cleaved form of PGAM5 was identified as a substrate of the inhibitors of apoptosis protein (IAPs), cellular guardians that are critical for controlling death-inducing proteins, such as caspases, Smac and HtrA2 [71]. IAPs contain both a RING ubiquitin ligase domain and characteristic baculoviral IAP repeat (BIR) domains that recognize substrates and promote their ubiquitination [72,73]. Interestingly, the *N*-terminal amino acid sequence of cleaved PGAM5 serves as a characteristic IAP binding motif (IBM) that is highly homologous to that of other classical IAP substrates [71]. Furthermore, the cleaved form of PGAM5 is found to accumulate in the cytosol during apoptosis and promote apoptosis by antagonizing IAPs.

On the other hand, PGAM5 has been shown to be involved in the induction of programmed necrosis, called necroptosis. Necroptosis is induced by the ligation of death domain-containing receptors, including TNF receptor 1 (TNFR1) and TNFR2, under specific conditions in which caspases are inhibited [74]. The main components of the necroptosis-signaling pathway are two Ser/Thr kinases, receptor-interacting protein 1 (RIP1) and RIP3 [75–77]. RIP3-mediated phosphorylation of PGAM5 enhances its protein phosphatase activity, and PGAM5, in turn, activates dynamin-related protein 1 (Drp1), a GTPase that is critically involved in mitochondrial fission, most likely through dephosphorylation of the inhibitory Ser637 site of Drp1 [78]. The resulting dephosphorylated Drp1 promotes mitochondrial fission and, thereby, induces necroptosis by still unknown mechanisms. The pro-necrotic role of PGAM5 may thus be exerted in part by the regulation of mitochondrial fission. In summary, PGAM5 appears to regulate apoptosis and necrosis positively and negatively in a manner highly dependent on cellular context, most likely including mitochondrial conditions.

## 5. Conclusions

The unique molecular and functional features of atypical protein phosphatases reviewed here reveal that protein phosphatases are more diverse than previously thought and strongly suggest that there are unique protein phosphatases whose structural features are beyond our current knowledge. Some findings implicating the involvement of atypical protein phosphatases in human diseases are important indicators that therapeutic targets for diseases that arise from dysregulation of protein dephosphorylation are not necessarily restricted to known protein phosphatases. Therefore, a broad range of research on the molecular functions of each atypical protein phosphatase, including identification of its substrates and an extensive exploration for new protein phosphatases, will expand our knowledge about protein phosphorylation and dephosphorylation and their physiological and pathophysiological significance.
